# RNA-binding protein MEX3D promotes cervical carcinoma tumorigenesis by destabilizing TSC22D1 mRNA

**DOI:** 10.1038/s41420-022-01049-7

**Published:** 2022-05-05

**Authors:** Zhi Zheng, Xiaojing Chen, Xiaoyun Cai, Hui Lin, Junfen Xu, Xiaodong Cheng

**Affiliations:** 1 Zhejiang Provincial Key Laboratory of Precision Diagnosis and Therapy for Major Gynecological Diseases, Women’s Hospital, Zhejiang University School of Medicine, 310006 Hangzhou, Zhejiang China; 2 Department of Obstetrics and Gynecology, Wenzhou People’s Hospital, 325000 Wenzhou, Zhejiang China; 3 Women’s Reproductive Health Laboratory of Zhejiang Province, Women’s Hospital, Zhejiang University School of Medicine, 310006 Hangzhou, Zhejiang China; 4 Department of Gynecologic Oncology, Women’s Hospital, Zhejiang University School of Medicine, 310006 Hangzhou, Zhejiang China

**Keywords:** Oncogenes, Predictive markers

## Abstract

RNA-binding proteins (RBPs) have been related to cancer development. Their functions in cervical cancer, however, are virtually unknown. One of these proteins, Mex-3 RNA-binding family member D (MEX3D), has been recently found to exhibit oncogenic properties in a variety of cancer types. In this present study, the functional roles and the regulatory mechanisms underlying MEX3D were examined in cervical cancer. The detection of MEX3D mRNA expression levels in cervical tissues was performed using reverse transcription-quantitative PCR. For functional analysis, for detecting apoptosis and cell proliferation in cervical cancer cells, the Cell Counting Kit-8, colony formation, and flow cytometry were utilized (SiHa and CaSki). The potential mechanisms of MEX3D were assessed and elucidated utilizing western blot analysis, RNA pull-down, RNA immunoprecipitation, and mRNA stability assays. For verification of MEX3D role in vivo, mouse xenograft models were established. When compared to normal cervical tissues, MEX3D expression was observed to be higher in cervical cancer tissues. MEX3D expression was increased in human papillomavirus (HPV) 16 positive cervical cancer tissues and positively regulated by HPV16 E7. When MEX3D expression was knocked down in cervical cancer cells, cell proliferation was decreased, colony formation was inhibited, and apoptosis was promoted. Furthermore, in a mouse xenograft model, knocking down MEX3D expression reduced cervical cancer tumor growth. In addition, MEX3D acted as an RBP to reduce TSC22 domain family protein 1 (TSC22D1) mRNA stability by directly binding to TSC22D1 mRNA. The findings revealed that MEX3D is upregulated by HPV16 E7 and has a crucial oncogenic in cervical cancer development via sponging TSC22D1 for destabilizing its mRNA levels. According to the findings of this study, MEX3D may be a potential therapeutic target for treating cervical cancer patients.

## Introduction

World’s fourth most prevalent malignancy in women is cervical cancer, with ~270,000 deaths and 530,000 new cases per year [1, 2]. Despite the effectiveness of HPV vaccination and well-organized screening in lowering cervical cancer incidence in developed countries, its usage in low-income countries is constrained, and progressive cervical cancer remains prevalent [3]. In total, low-income countries account for ~90% of world’s cervical cancer-related mortalities [4–6]. With a 5-year survival rate of only 16.7% for patients with progressive cervical cancer, the curative effect is suboptimal [7]. Therefore, the identification of novel molecular aberrations that lead to cervical cancer development may be used to achieve optimal therapeutic management for this malignancy.

RNA-binding proteins (RBPs) are critical for the transcriptome as they have the ability to modulate practically every step of the post-transcriptional process, from alternative splicing to stability and decay of RNA [8]. Hence, RBPs dysfunction affects practically every phase of cancer formation and progression, including angiogenesis stimulation, cell proliferation, immune surveillance evasion, invasion, metastasis activation and apoptosis resistance [9].

The Mex-3 RNA-binding family member D (MEX3D) gene functions as an RBP and is one of four human homologous MEX-3 genes MEX-3 is a K-homology (KH) domain-containing RBP, which was initially defined as a translational repressor in Caenorhabditis elegans. In human and mouse genus, four MEX3 homologous genes have been discovered: MEX3A, MEX3B, MEX3C, and MEX3D. These genes encode for enzymes with E3 ubiquitin ligase activity mediated by a ring finger domain at the C terminal. They are also considered critical for RNA degradation [10, 11]. The ring finger domain confers the human MEX3 protein the potential to modulate target protein ubiquitination, hence affecting their protein stability and subcellular localization [10, 12, 13]. MEX3A has been associated with cancers development, including glioma, gastric cancer, and breast cancer, according to studies [14–17]. MEX3B was reported to enhance the invasion of gastric cancer cells [18]. MEX3C was shown to promote osteosarcoma malignant progression and bladder tumorigenesis [19, 20]. Previous studies have shown that MEX3D is an oncogenic driver in prostate cancer [21, 22]. In cervical cancer, the role and expression of MEX3D, however, are poorly understood. In our previous RNA sequencing (RNA-seq) results, in cervical cancer tissues, MEX3D mRNA expression was elevated compared to the corresponding expression noted in normal cervical tissues [23]. The intention of this study is determining MEX3D the significant role in cervical cancer. The RNA-binding protein MEX3D role as a tumor promoter in cervical cancer was assessed in this study. MEX3D effect on cell viability, colony formation, and cell apoptosis was investigated in cervical cancer cells. Subsequently, HPV16 oncogene E7 influence on MEX3D expression was evaluated. The data indicated that MEX3D was associated with TSC22 domain family protein 1 (TSC22D1) RNA levels and that TSC22D1 knockdown could reverse MEX3D knockdown-induced tumorigenesis in cervical cancer cells. The current study presented a theoretical basis for using this target in the management of patients with cervical cancer and identified a new post-transcriptional mechanism involving MEX3D-mediated TSC22D1 transcript destabilization.

## Results

### MEX3D expression is frequently upregulated in cervical cancer tissues

**Fig. 1:**
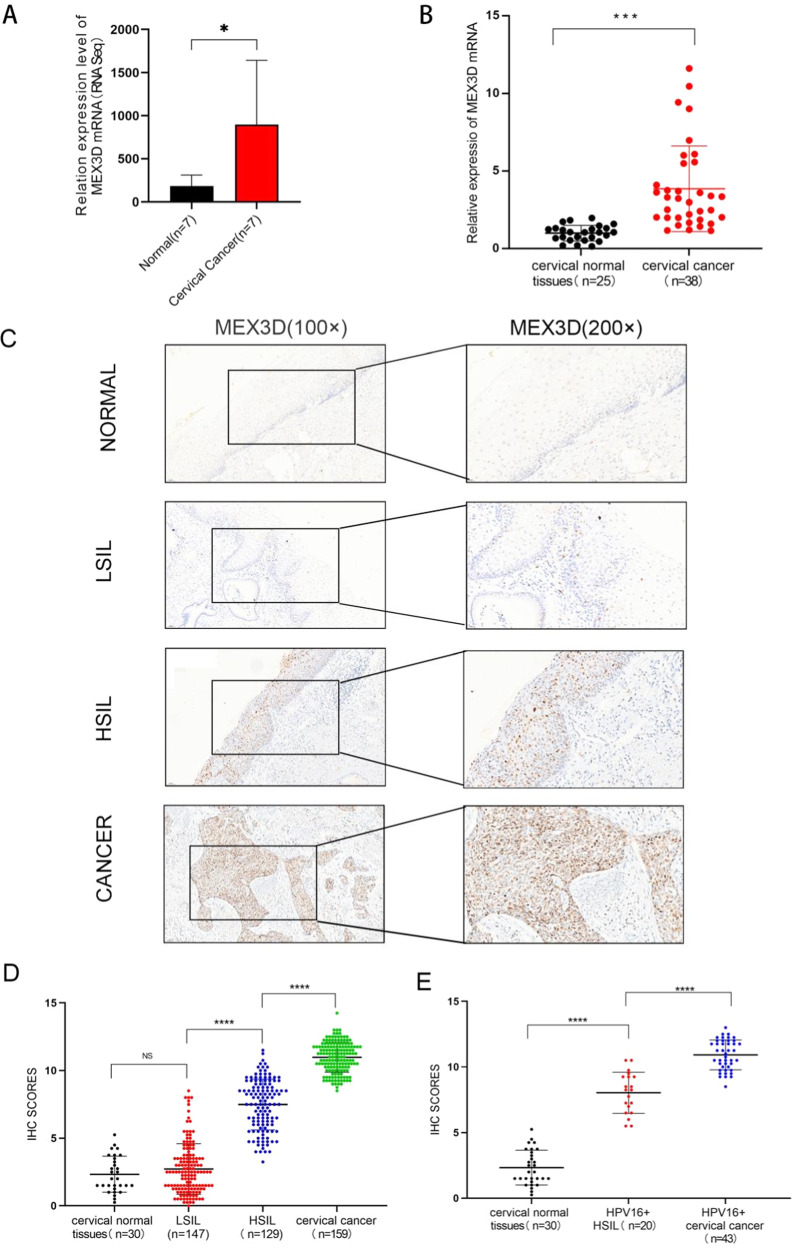
MEX3D expression is elevated in cervical cancer tissues. **A** RT-qPCR validation of MEX3D mRNA in seven cervical cancer tissues and seven normal cervical tissues. **B** MEX3D mRNA expression levels in 25 cervical normal epithelium samples and 38 cervical cancer tissue samples. **C** Representative IHC images of MEX3D expression of the normal cervix, LSIL, HSIL, and cervical cancer tissues. **D** IHC scores analyses of MEX3D protein expression in normal cervix (*n* = 30), LSIL (*n* = 147), HSIL (*n* = 129), and cervical cancer tissues (*n* = 159). **E** IHC scores analyses of MEX3D protein expression in HPV16-negtive normal cervix (*n* = 30), HPV16-positive HSIL (*n* = 20), and HPV16-positive cervical cancer (*n* = 43). NS not significant; *P < 0.05, ***P < 0.001, ****P < 0.0001.

MEX3D mRNA was noticed to be substantially elevated in cervical cancer tissues than the corresponding expression noticed in normal cervical tissues in our prior RNA-seq data (Fig. 1A) [23]. To confirm our sequencing data, reverse transcription-quantitative PCR was utilized for measuring MEX3D mRNA expression levels in an additional cohort of 25 normal cervical tissues and 38 human cervical cancer tissues. In cervical cancer tissue specimens, MEX3D mRNA expression levels were significantly elevated than in normal cervix specimens (Fig. 1B). Following that, the MEX3D protein expression level was assessed in 465 cervical tissues, comprising 30 normal cervix tissues, 147 low-grade squamous intraepithelial lesions (LSIL), 129 high-grade squamous intraepithelial lesions (HSIL), and 159 cervical cancer tissues. Immunohistochemistry (IHC) was used for the analysis. The MEX3D protein expression level in normal and LSIL tissues showed no significant differences. It is interesting to note that MEX3D protein overexpression was noted in HSIL and cervical cancer samples compared to LSIL or normal tissues. Furthermore, MEX3D protein expression was considerably greater in cervical cancer tissues (Fig. 1C, D). To examine whether High-risk HPV infection influences the altered expression of MEX3D protein, IHC scores of cervical tissues with or without HPV16 infection were studied further. The following results were found: MEX3D protein level was increased in HPV16-positive HSIL compared to HPV16 negative normal tissues. Similarly, expression level of MEX3D protein was higher in HPV16-positive cervical cancer than in HPV16-positive HSIL tissues. As depicted in Fig. 1E. The findings suggested that MEX3D, as an oncogene, may have a role in cervical cancer. HPV16 infection may lead to increased expression of MEX3D.

### MEX3D inhibits cervical cancer cells apoptosis and promotes proliferation

**Fig. 2:**
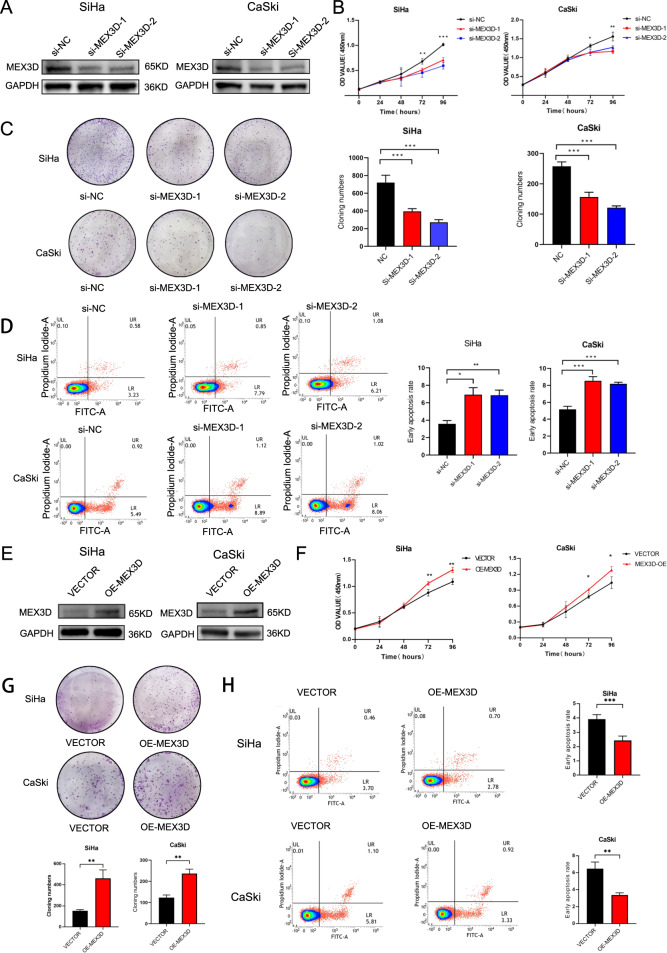
MEX3D stimulates cervical cancer cells proliferation whereas inhibits its apoptosis. **A** Western blot analysis was utilized for estimating MEX3D protein expression levels in CaSki and SiHa cells after being transfected with a negative control siRNA or two MEX3D-specific siRNAs. **B** The growth curve in CaSki and SiHa cells was estimated through CCK8 assays after being transfected with a negative control siRNA or two MEX3D-specific siRNAs. **C** Knockdown of MEX3D with two MEX3D-specific siRNAs suppressed cell viability shown by colony formation in CaSki and SiHa cells. **D** Apoptosis level in CaSki and SiHa cells after being transfected with two MEX3D-specific siRNAs or a negative control siRNA. **E** Western blot was utilized for determining the levels of MEX3D overexpression in CaSki and SiHa cells utilizing a constructed and control plasmid. **F**–**H** MEX3D overexpression stimulated cellular proliferation (**F**, **G**) whereas the suppressed rate of cellular apoptosis in CaSki and SiHa cells (**H**). *P < 0.05, **P < 0.01, ***P < 0.001.

Considering that uncontrolled cell proliferation is a hallmark of cancer [24, 25], this study evaluated whether inhibiting MEX3D suppressed the proliferation of cervical cancer cells. Two sequences of MEX3D-specific small interfering RNA (siRNA) were utilized to eliminate MEX3D expression in CaSki and SiHa cervical cancer cells. MEX3D protein expression knockdown reduced cell proliferation and promoted cellular apoptosis in two cell lines. (Supplementary Fig. S1A, B and Fig. 2A–D). Subsequently, the role of MEX3D overexpression was investigated in CaSki and SiHa cells utilizing a constructed plasmid for MEX3D expression upregulation. MEX3D overexpression increased cellular proliferation while suppressing apoptosis (Supplementary Fig. S1C and Fig. 2E–H). Taken together, these data reinforce the hypothesis suggesting that MEX3D serves as a tumor promoter in cervical cancer progression and facilitates the malignant phenotype of cervical cancer.

### MEX3D is associated with TSC22D1 RNA levels and regulates the stability of TSC22D1 mRNA in cervical cancer cells

**Fig. 3:**
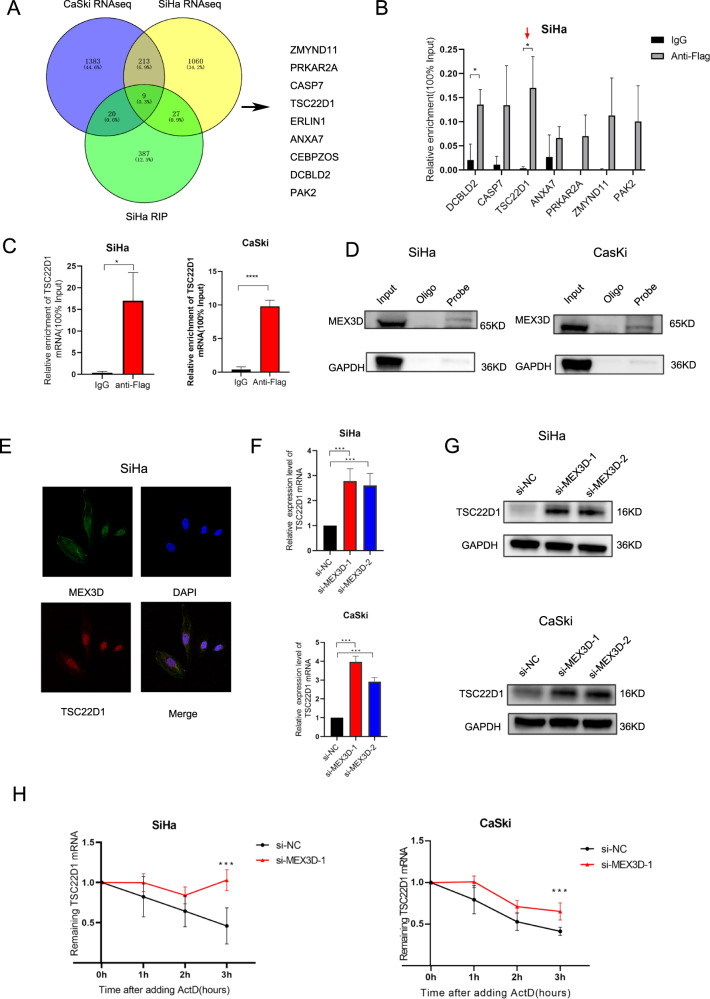
MEX3D is related to TSC22D1 RNA levels and regulates TSC22D1 mRNA stability in cervical cancer cells. **A** Venny diagram of transcripts stabilized and bound by MEX3D. Transcripts bound by MEX3D in the RIP sequencing data and transcripts destabilized upon MEX3D knockdown in CaSki and SiHa cells. **B** The mRNA levels of seven genes were determined utilizing qRT-PCR in SiHA cells overexpressing MEX3D. When comparing the anti-flag sample to the IgG sample, TSC22D1 mRNA was substantially enriched. **C** TSC22D1 mRNA was found to bind to MEX3D and confirmed by RIP assay both in CaSki and SiHa cells. **D**, **E** For validating the interactions of MEX3D with TSC22D1, RNA pull-down and RNA-fluorescent in situ hybridization assays were done. **F**, **G** Downregulation of MEX3D expression in CaSki and SiHa cells elevated TSC22D1 mRNA and protein levels. **H** To inhibit RNA synthesis, actinomycin-D was added. TSC22DQ mRNA levels in CaSki and SiHa cells with MEX3D were knockdown or not. *P < 0.05, ***P < 0.001, and ****P < 0.0001.

In cervical cancer, the underlying molecular mechanism of the MEX3D cancer-promoting effects was investigated. Given that MEX3D is a member of the RBP family, the present study aimed to identify its direct targets and its biological effects. Initially, the identification of the endogenous RNA targets of MEX3D was performed in cervical cancer cells by high-throughput RNA immunoprecipitation (RIP) sequencing (analyzed by RiboBio, Guangzhou, China). SiHa cervical cancer cells were used, which were expressing Flag-tagged MEX3D. In addition, RNA-seq analysis (analyzed by GeneChem, Shanghai, China) was conducted to affirm the down-/upregulation of the expression levels of specific genes identified in MEX3D knockdown cells. Via these analyses, we identified nine genes (DCBLD2, CASP7, TSC22D1, ANXA7, PRKAR2A, ZMYND11, CEBPZOS, ERLIN1, and PAK2) (Fig. 3A). In addition, DCBLD2, CASP7, TSC22D1, ANXA7, PRKAR2A, ZMYND11, and PAK2 have been reported to be tightly linked to tumor malignancy in previous studies [26–32]. Therefore, they were identified as possible MEX3D targets in cervical cancer. Subsequently, MEX3D overexpressing SiHa cells were constructed, which were transfected with a Flag-label-inserted plasmid. The TSC22D1 mRNA levels were significantly enriched in anti-flag samples compared with the corresponding expression noted in immunoglobulin G samples derived from SiHa cells (Fig. 3B). TSC22D1 mRNA was bound to MEX3D. This binding was confirmed in CaSki and SiHa cells utilizing RIP assay (Fig. 3C). The RNA pull-down and RNA-fluorescent in situ hybridization assays were conducted to further confirm the interactions between MEX3D and TSC22D1 (Fig. 3D, E). Furthermore, downregulation of MEX3D expression in CaSki and SiHa cells increased TSC22D1 mRNA and protein levels (Fig. 3F, G). Considering that one of the most important functions of RBPs is the regulation of RNA stability, enhanced stability of TSC22D1 mRNA was found in SiHa and CaSki cells following MEX3D knockdown (Fig. 3H).

### Knockdown of TSC22D1 expression attenuates MEX3D-mediated tumorigenesis

**Fig. 4:**
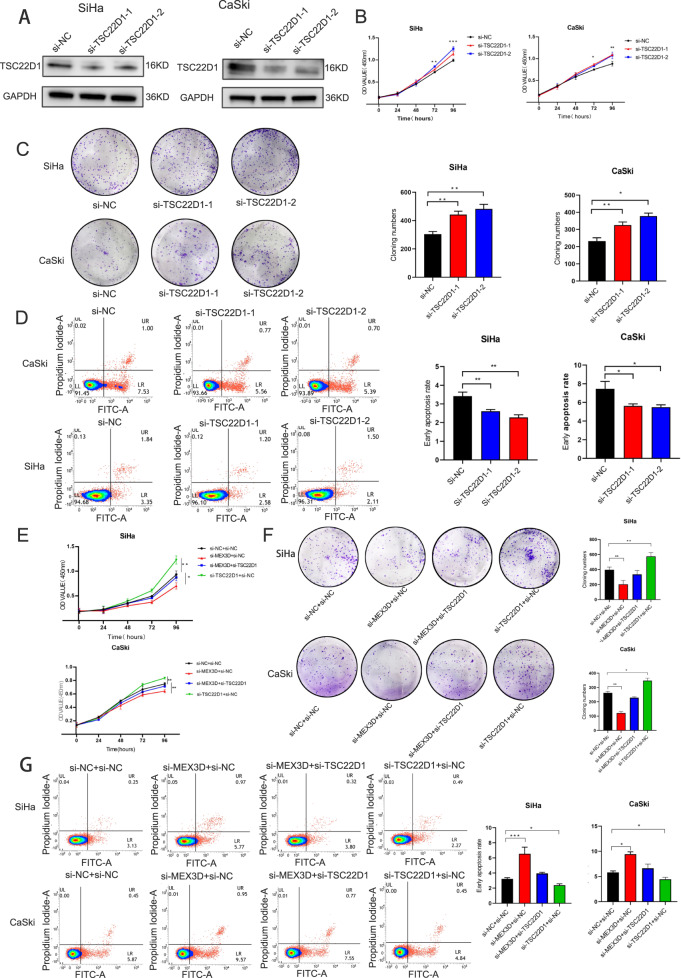
TSC22D1 knockdown attenuates MEX3D-mediated tumorigenesis. **A** TSC22D1 knockdown significantly by siRNAs in protein levels in CaSki and SiHa cells. **B**–**D** TSC22D1 knockdown stimulated cellular proliferation, whereas the suppressed rate of cellular apoptosis. **E**–**G** TSC22D1 knockdown mitigated the decreased cell proliferation and increased cellular apoptotic rate mediated by MEX3D knockdown. *P < 0.05, **P < 0.01, and ***P < 0.001.

Given that TSC22D1 is a key MEX3D downstream effector, the current study characterized the TSC22D1 functional role in tumorigenesis of cervical cancer. In CaSki and SiHa cells, knockdown of TSC22D1 expression with two different sequences of siRNA was performed (Supplementary Table S2, Supplementary Fig. S1D, E, and Fig. 4A). Knockdown of TSC22D1 expression suppressed the cellular apoptotic rate and enhanced cellular proliferation (Fig. 4B–D). A rescue experiment was carried out to see if TSC22D1 was involved in the consequences of MEX3D-mediated carcinogenesis in cervical cancer cells. Simultaneous knockdown of MEX3D and TSC22D1 expression was performed in SiHa and CaSki cells. TSC22D1 knockdown partially mitigated the lower cell proliferation capacity and increased cellular apoptotic rate caused by MEX3D knockdown, according to the findings (Fig. 4E–G). TSC22D1 was found to be a critical inhibitory factor in the carcinogenesis of cervical cell, and TSC22D1 is essential for reducing the tumorigenic potential of cervical cancer cells induced by MEX3D.

### Knockdown of MEX3D expression reduces xenograft tumor growth

**Fig. 5:**
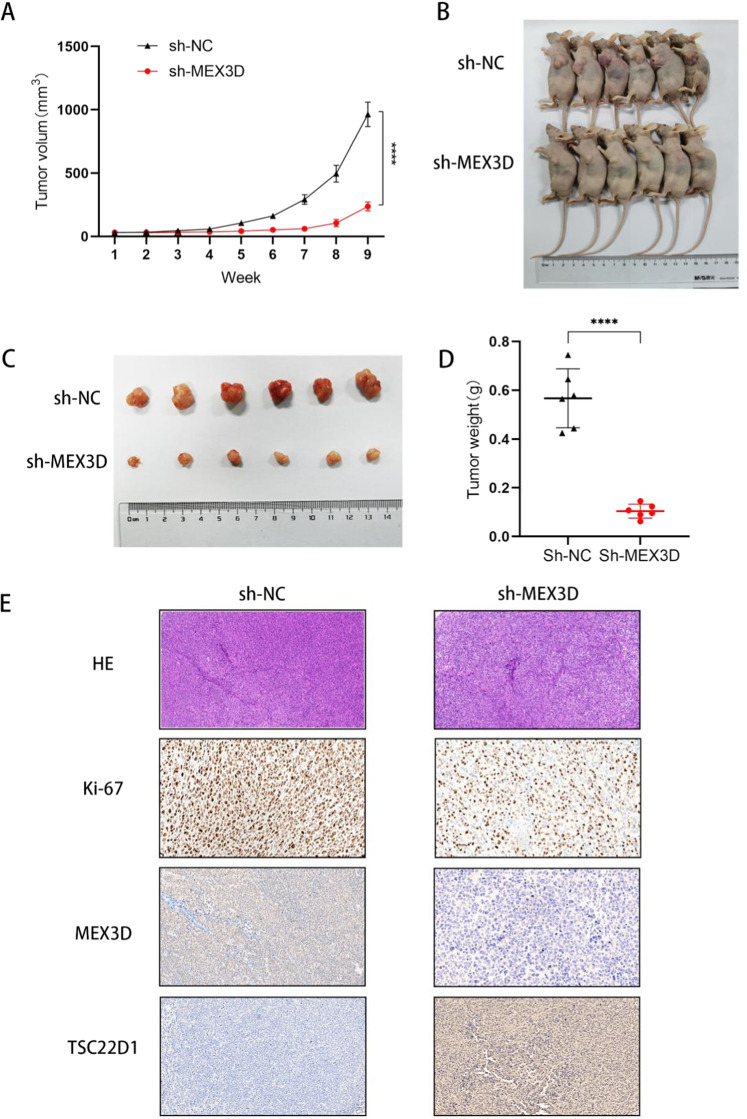
MEX3D expression knockdown reduces the growth of xenograft tumor. Non-SCID mice were injected with SiHa/NC-shRNA and SiHa/MEX3D-shRNA cells subcutaneously. Tumor volume was estimated every 7 days, and all mice were euthanized under general anesthesia 9 weeks following injection. **A** SiHa/MEX3D-shRNA cells grow at a significantly slower rate than SiHa/NC-shRNA cells. **B** Representative images of mice euthanized at week nine following injection (*n* = 6). **C** Macro images of subcutaneous tumors. **D** Subcutaneous tumor weights in naked mice using sh-MEX3D or a negative control. **E** H&E, Ki-67, MEX3D, and TSC22D1 protein expression in xenograft tumors were analyzed by IHC. ****P < 0.0001.

Following the investigation of the function of MEX3D in vitro, animal experiments were carried out to confirm the findings in vivo. Subcutaneous transplantation of SiHa/MEX3D-short hairpin RNA (shRNA) and SiHa/negative control (NC)-shRNA cells into non-SCID mice was done. SiHa/MEX3D-shRNA cell growth was significantly lower than SiHa/NC-shRNA cell growth (Fig. 5A). In addition, both the tumor size and weight of the animals injected with SiHa/MEX3D-shRNA cells were lowered significantly compared to the tumors obtained through the SiHa/NC-shRNA cells (Fig. 5B–D). H&E staining was conducted to confirm tumorigenesis (Fig. 5E). To establish if MEX3D was capable of regulating cell proliferation in vivo, the Ki-67 antigen expression levels (a well-known cellular proliferation markers) were estimated utilizing IHC. As listed in Fig. 5E, in tumors obtained through SiHa/MEX3D-shRNA cells, a smaller number of Ki-67 antigen-positive cells was observed than in tumors obtained through SiHa/NC-shRNA cells. In addition, this revealed that MEX3D expression knockdown might inhibit cervical cancer cell proliferation.

### HPV16 E7 in cervical cancer cells promotes MEX3D expression

**Fig. 6:**
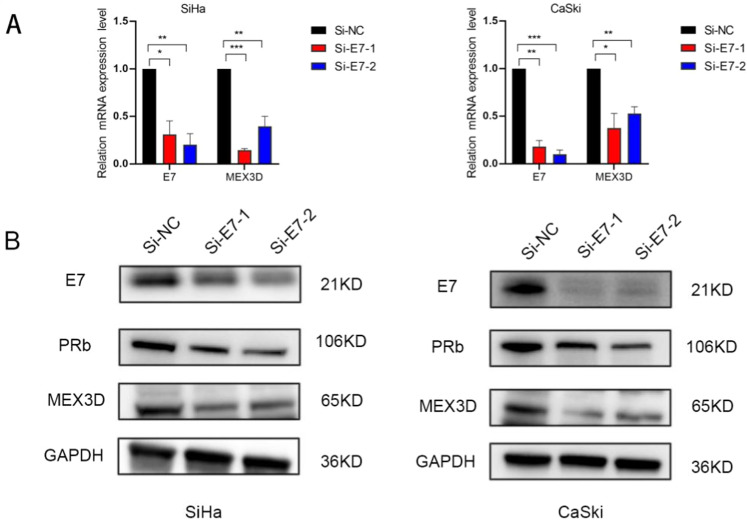
HPV16 E7 stimulates MEX3D expression in cervical cancer cells. **A** Levels of MEX3D mRNA expression were analyzed by qRT-PCR in SiHa and CaSki cells underwent transfection with two E7 siRNAs and si-NC. **B** Protein levels of MEX3D, E7 and pRb were determined following transfection with two E7 siRNAs and si-NC. *P < 0.05, **P < 0.01, and ***P < 0.001.

HPV16 oncoprotein E6/E7 are critical in tumor cell growth. We further investigated whether the increased expression of MEX3D in cervical cancer was regulated by E6 or E7. We first treated CaSki and SiHa cells expressing high levels of HPV16 E6/E7 with HPV16 E6 siRNA. There was no change observed in the expression of MEX3D mRNA following HPV16 E6 knockdown (Supplementary Fig. S1F, G). Then HPV16 E7 siRNA sequences underwent incubation in CaSki and SiHa cells to examine if MEX3D is implicated in the tumor-promoting properties of HPV16 E7. MEX3D mRNA and protein levels were significantly lowered by HPV16 E7 knockdown (Fig. 6A, B). As a result of these findings, it was suggested that HPV16 oncoprotein E7 is capable of upregulating MEX3D expression.

## Discussion

The function of the RBP MEX3D and its mechanism of action in cervical cancer were highlighted in this work. Alterations in RNA processing events have a role in pathophysiology of cancer. In RNA processing, RBPs interact mainly with RNA transcripts to form ribonucleoprotein complexes and influence RNA quality via splicing, polyadenylation, nuclear export, protein translation modification, and RNA transcripts decay [33, 34]. It has been shown that RBPs play tumor-suppressive or oncogenic roles that affect the development of various cancer types, comprising breast cancer, lung cancer, and ovarian cancer. This indicated RBPs significant roles in the progression of cancer and can be utilized as potential therapeutic targets for cancer treatment [35–38]. Even so, RBPs’ biological function and potential utility in cervical cancer are obscure. In this study, the RBP MEX3D was assessed with regard to its oncogenic role in cervical cancer. MEXED was directly bound to TSC22D1 mRNA and reduced its instability. These findings demonstrated the necessity of post-transcriptional regulation and RBPs in the development of cervical cancer.

MEX3D was originally described as a member of the MEX3 gene family (MEX3A-D) [10]. Shao et al. reported that MEX3D functioned as an oncogene in prostate cancer [22]. Even so, the MEX3D gene function in cancer is poorly characterized. To the best of our knowledge, MEX3D has never been linked to cervical cancer. MEX3D expression was higher in human cervical cancer cells, suggesting that it may be a strong predictor for cervical cancer tumorigenesis in this study.

**Fig. 7:**
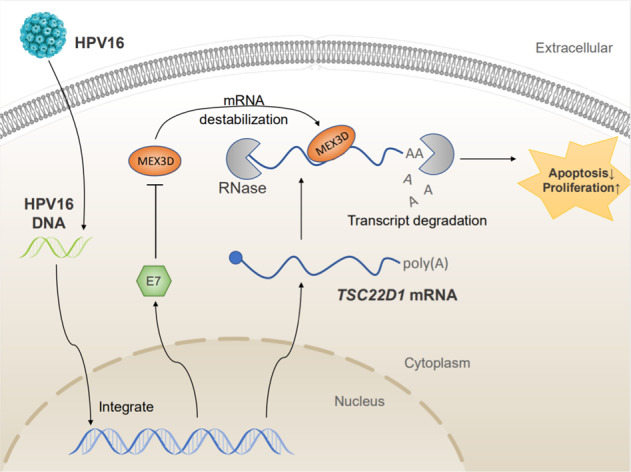
This article schematic diagram. Sufficient quantities of endogenous MEX3D generated during cervical cancer tumorigenesis are demonstrated by schematic diagram. HPV16 E7 activates MEX3D expression in cervical cancer cells. MEX3D binds to the TSC22D1 mRNA and destabilizes TSC22D1 mRNA and promoting proliferation and reducing apoptosis of cervical cancer.

It is generally considered that cervical cancer is a complex pathological process during which cervical cancer cells undergo proliferation and apoptosis. In this study, it was confirmed that MEX3D acted as a functional protein that promoted proliferation. Knocking down MEX3D expression decreased cervical cancer growth and promoted apoptosis in both CaSki and SiHa cells. In addition, MEX3D overexpression induced cervical cancer cell proliferation and attenuated apoptosis. Therefore, the data indicated that high MEX3D expression acted as a tumor promoter in cervical cancer progression (Fig. 7).

Human TSC22D1 is a candidate suppressor, and its expression has been confirmed in various cancer types, such as malignancies of the salivary glands, prostate, and brain [28, 39–42]. The mechanism of TSC22D1 in cervical cancer, however, is unknown. In accordance with past findings, this study identified that knockdown of TSC22D1 expression significantly promoted cell proliferation and reduced induction of cell apoptosis. In addition, knockdown of TSC22D1 expression partially neutralized cervical cell tumorigenesis induced by MEX3D.

HPVs have been defined as the causal agents of cervical cancer, with HPV-positive cervical malignancies, HPV16 is the commonest isolated type [43, 44]. Two viral oncoproteins, namely E6 and E7, are considered to contribute to tumor progression by inactivating the tumor suppressor genes p53 and retinoblastoma (pRb) [45, 46]. The ability of the HPV E7 protein to bind to pRb is a critical function, leading to altered activity of this cell-cycle regulator [47]. In this study, HPV16 E7 expression knockdown caused a dramatic reduction in the expression levels of MEX3D. The data indicated that the MEX3D/TSC22D1 complex regulated cancer-associated functions as a consequence of HPV16 E7 overexpression.

In conclusion, The RBP MEX3D expression levels were elevated by HPV16 E7, according to the present research. MEX3D played a vital role by reducing TSC22D1 mRNA stability in apoptosis and tumor growth. Therefore, the present study demonstrated that in cervical cancer, MEX3D could be utilized as therapeutic target and potential diagnostic.

## Materials and methods

### Clinical samples

The Women’s Hospital of Zhejiang University’s Ethical Committee accepted the study (IRB-20200263-R). Prior to sample collection, each subject who underwent surgical resections signed a written informed permission form. The trials were carried out in compliance with the instructions that had been approved. From January 2013 to December 2018, at Women’s Hospital, Zhejiang University School of Medicine’s, all human cervical tissues were acquired. Two senior pathologists affirmed all tissue samples pathologically and clinically. Supplementary Table S1 comprises the patient data for all paraffin sections.

### RNA extraction and qRT-PCR

In compliance with the manufacturer’s instructions, TRIzol reagent (Invitrogen, Carlsbad, CA, USA) was utilized for RNA extraction. The SYBR Premix Ex Taq (TaKaRa, Japan) and PrimeScript RT Reagent Kit were utilized for conducting qRT-PCR experiments. All of the primers utilized in this study are listed in Supplementary Table S2.

### Cell culture

The American Type Culture Collection was utilized to provide the HPV16-positive human cervical carcinoma cell line SiHa (ATCC, USA). Another HPV16-positive human cervical cancer cell line CaSki, was retrieved from the Cell Resource Center at the Shanghai Institute of Life Sciences of the Chinese Academy of Sciences (China), where it was authenticated and tested. SiHa cells underwent incubation in DMEM (BI, Israel) with 10% FBS (Everyday Green, Hangzhou, China) at 37 °C with 5% CO_2_. Whereas CaSki underwent incubation in RPMI-1640 (BI, Israel) supplemented with 10% FBS.

### Western blotting

Lysis buffer was used to extract cellular proteins. Using 10% SurePAGE Egels (GenScript, US), 20 μg of total protein was segregated and transmitted to PVDF membranes (Bio-Rad, USA, 1620177). In compliance with their manufacturer’s guidelines, Western blot analysis was carried out utilizing the indicated antibodies. In this study, the following antibodies were utilized: anti-MEX3D (Santa Cruz, USA), anti-E7 (Santa Cruz, USA), anti-GAPDH (Diagbio, China), anti-TSC22D1 (Proteintech, China), anti-pRb (Santa Cruz, USA). Each trial was conducted three times independently.

### Gene knockdown and overexpression

MEX3D small interfering RNAs (siRNA) were generated by GenePharma (Shanghai, China). HPV16 E6 siRNA, HPV16 E7 siRNA, and TSC22D1 siRNAs were designed by RiboBio (Guangzhou, China). DharmaFECT Transfection Reagents were utilized for conducting siRNAs transfection (Thermo, USA). Lentiviral vector GV493 (GeneChem, Shanghai, China) was used to introduce short hairpin RNAs (shRNAs) and transduced them into SiHa cells following the instructions. Puromycin resistance was used to obtain stable cell lines. The MEX3D overexpression plasmid was constructed by cloning the full-length MEX3D cDNA into the CMV-MCS-3FLAG-SV40-Purocycin vector (GeneChem, Shanghai, USA). X-treme GENE HP DNA Transfection Reagent was utilized to transfect the plasmids (Roche, China). Supplementary Table S2 lists the shRNA and siRNA target sequences, as well as the primer sequences utilized in this study.

### CCK8 assay

In 96-well plates, 4000 SiHa and CaSki cells were planted. The CCK8 assay was done at 24, 48, 72, and 96 h after being transfected (Dojindo, Minato-ku, Tokyo, Japan). A spectrophotometer reader at 450 nm measured the absorbance. Each trial was conducted three times independently.

### Colony-formation assays

The ability of cervical cancer cells to clone was tested using colony-formation tests. For colony-formation experiments, treated SiHa and CaSki cells (1000 cells/well) were incubated for 14 days and planted into six-well plates. At room temperature, the colonies were dyed with a 0.05% crystal violet solution for 30 min. The images were taken with a Canon camera. Each experiment was repeated three times.

### Apoptosis assay

At 72 h following transfection, the rate of apoptosis of treated CaSki and SiHa cells was evaluated utilizing an Annexin V-FITC/PI Apoptosis Kit (Mutisiences, China, AP101–100-kit). Cells were resuspended after rinsing with PBS in a binding buffer containing Annexin V-FITC and PI. After 15 min of incubation, a flow cytometer (BD Biosciences) were utilized for investigating cells apoptosis rate. Each trial was conducted three times independently.

### RNA pull-down

Pierce™ Magnetic RNA-Protein Pull-Down Kit (Thermo Scientific, USA) was utilized for conducting RNA pull-down assays. Biotin-labeled RNA probes were designed at singlemoleculefish.com and synthesized by TsingKe (China). Sequence of TSC22D1 sense probe was 5′-agtgtcttcagcagattgtt-3′ and sequence of oligo probe was 5′-aacaatctgctgaagacact-3′. For 30 min, streptavidin magnetic beads underwent incubation at room temperature with TSC22D1 sense and oligo probes. After washing three times at 4 °C, the beads were coupled with proteins in RNA-Protein Binding Buffer. Western blot analysis was utilized to elute proteins linked to beads.

### RNA immunoprecipitation (RIP)

In compliance with the manufacturer’s instructions, EZ-Magna RIP kit (Millipore, USA) was utilized for performing RIP analysis. In the experiment, an antibody specific for flag (MultiSciences, China) and isotype control antibody (IgG) were utilized. Protein A/G magnetic beads underwent incubation with antibodies for 30 min at room temperature. The beads underwent incubation at 4 °C overnight with cell lysates after being washed three times with RIP wash buffer. qRT-PCR was used to detect the co-precipitated RNA. The input % was utilized for calculating Flag’s relative expression.

### Fluorescence in situ hybridization (FISH)

The RiboTM Fluorescent In Situ Hybridization Kit (RiboBio, China) was utilized to perform FISH as directed with Cy3-labeled probes. A confocal microscope was utilized for visualizing In situ hybridization signals (TCS SP2 AOBS). CY3-labeled TSC22D1 probe was designed at singlemoleculefish.com and synthesized by Tsingke, China (illustrated in Supplementary Table S2). Anti-MEX3D antibody was purchased from Santa Cruz, USA.

### RNA stability analysis

For evaluating the TSC22D1 mRNA half-life, actinomycin-D (5 μg/ml, Sigma, A4262) was added for mRNA synthesis blockage. At various time points, total RNA was isolated and subjected to RT-qPCR analysis. TSC22D1 mRNA levels were standardized to GAPDH and plotted as a percentage of the value at the time of the addition of actinomycin-D.

### Nude mice xenograft experiments

Lentiviral vectors expressing shRNA targeting MEX3D were used to infect SiHa cells. SiHa/MEX3D-shRNA and NC-shRNA (infected cells) (8 × 10^6^) were subcutaneously injected into 4-week-old female non-SCID mice (*n* = 6 in each group) in the right-side flank area. Every 7 days following injection, tumors were estimated utilizing precision callipers, and the volume of tumor was determined utilizing the formula [(tumor length) × (tumor width) × (tumor width)]/2. Following injection, at proper time points, all animals were slaughtered. We weighed, excised, and photographed subcutaneous tumors. After being fixed overnight in 10% paraformaldehyde, for further investigation, the tumor tissues were sectioned and embedded in paraffin. All animal tests were undertaken in compliance with the animal guidelines for utilization and laboratory animal care, which were authorized by Zhejiang Chinese Medical University’s Animal Ethical and Welfare Committee (IACUC-20201207-07).

### Immunohistochemistry

Utilizing the MEX3D antibody (Abcam, China, 1:600) on paraffin-embedded cervical tissue segments, immunohistochemistry (IHC) was done. Protein immunohistochemical expression was determined utilizing a scoring system depending on the staining range and product of staining intensity. The staining intensity was graded as 0 (no staining), 1 (weak staining), 2 (moderate staining), and 3 (strong staining). The staining range was graded as 0 (0% stained), 1 (1–25% stained), 2 (26–50% stained), 3 (51–75% stained), and 4 (76–100% stained). In four consecutive high-magnification fields (×200), surface area scores and staining intensity were multiplied for generating an expression score. After then, the average value was calculated. By multiplication of these two values, the final product score was estimated, resulting in a range of 0 to 12.

### Statistical analysis

All statistical analyses were conducted utilizing GraphPad Prism 9.0 Software (GraphPad Software, USA) and SPSS Statistics 20.0 (IBM, USA). Data in compliance with normal distribution were expressed as mean standard ± deviation, and data across two groups were estimated utilizing Student’s *t t*ests. Besides that, the data were presented as median ± interquartile range, and Mann–Whitney tests were utilized. Differences with *P* < 0.05 were deemed statistically significant.

## Supplementary information

Supplementary Table S1

Supplementary Table S2

Supplementary Figure1

Original Data File

## Data Availability

All data generated or analyzed in the paper are included in this article.
