# Supplementary material for: RNA-binding protein MEX3D promotes cervical carcinoma tumorigenesis by destabilizing TSC22D1 mRNA
Source: Cell Death Discov. 2022 May 5;8:250. doi: 10.1038/s41420-022-01049-7 (PMC9072549; doi:10.1038/s41420-022-01049-7)
Supplement: Supplementary file 3 — Supplementary Figure1 [file 41420_2022_1049_MOESM3_ESM.docx]

**Supplementary Fig. 1**

**
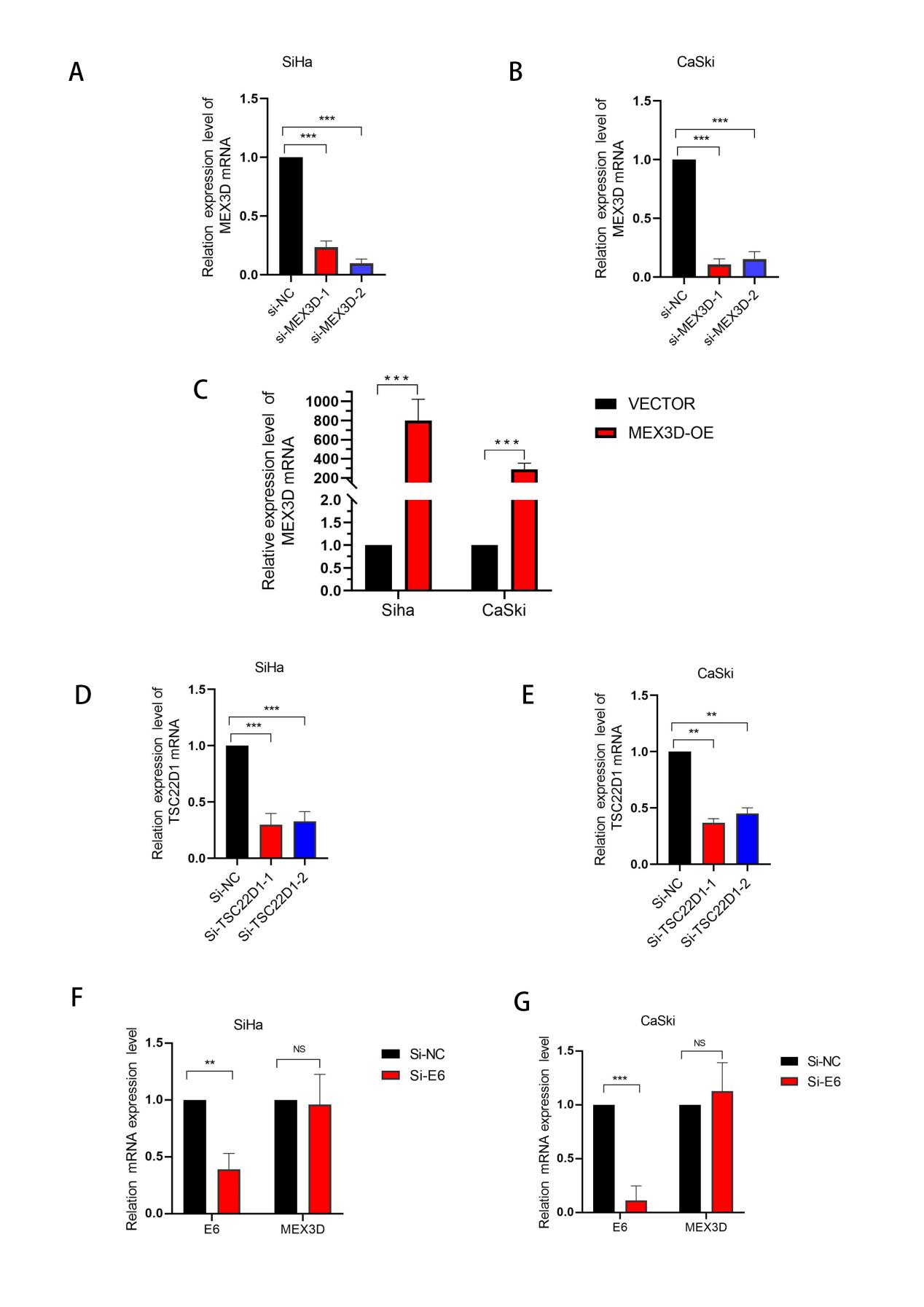
**

**Supplementary Fig. 1 a, b** RT-qPCR was utilized for estimating MEX3D mRNA expression levels in CaSki and SiHa cells after being transfected with a negative control siRNA or two MEX3D-specific siRNAs. **c** The mRNA levels of MEX3D overexpression in CaSki and SiHa cells utilizing a constructed and control plasmid. **d, e** TSC22D1 knockdown significantly by two TSC22D1-specific siRNAs in mRNA levels in SiHa and CaSki cells. **F, g** MEX3D mRNA levels were determined by qRT-PCR in SiHa and CaSki cells following transfection with si-E6 and si-NC. NS, not significant; ***P* < 0.01, ****P* < 0.001, *****P* < 0.0001.
