# Supplementary material for: RNA-binding protein MEX3D promotes cervical carcinoma tumorigenesis by destabilizing TSC22D1 mRNA
Source: Cell Death Discov. 2022 May 5;8:250. doi: 10.1038/s41420-022-01049-7 (PMC9072549; doi:10.1038/s41420-022-01049-7)
Supplement: Supplementary file 4 — Original Data File [file 41420_2022_1049_MOESM4_ESM.docx]

**Original Western Blots**





**Fig. 1** MEX3D protein expression levels in SiHa cells after being transfected with a negative control siRNA or two MEX3D-specific siRNAs.





**Fig. 2** GAPDH protein expression levels in SiHa cells after being transfected with a negative control siRNA or two MEX3D-specific siRNAs.





**Fig. 3** MEX3D protein expression levels in CaSki cells after being transfected with a negative control siRNA or two MEX3D-specific siRNAs.





**Fig. 4** GAPDH protein expression levels in CaSki cells after being transfected with a negative control siRNA or two MEX3D-specific siRNAs.





**Fig. 5** levels of MEX3D overexpression in SiHa cells utilizing a constructed and control plasmid.





**Fig.6** levels of GAPDH protein expression levels in SiHa cells utilizing a constructed and control plasmid.





**Fig. 7** levels of MEX3D overexpression in CaSki cells utilizing a constructed and control plasmid.





**Fig. 8** levels of GAPDH protein expression levels in CaSki cells utilizing a constructed and control plasmid.





**Fig. 9** levels of MEX3D protein expression levels in SiHa cells utilizing RNA pull-down assays.





**Fig. 10** levels of GAPDH protein expression levels in SiHa cells utilizing RNA pull-down assays.





**Fig. 11** levels of MEX3D protein expression levels in CaSki cells utilizing RNA pull-down assays.





**Fig. 12** levels of GAPDH protein expression levels in CaSki cells utilizing RNA pull-down assays.





**Fig. 13** Downregulation of MEX3D expression in SiHa cells elevated TSC22D1 protein levels.





**Fig. 14** levels of GAPDH protein expression after downregulation of MEX3D expression in SiHa cells.





**Fig. 15** Downregulation of MEX3D expression in CaSki cells elevated TSC22D1 protein levels.





**Fig. 16** levels of GAPDH protein expression after downregulation of MEX3D expression in CaSki cells.





**Fig. 17** TSC22D1 protein expression levels in SiHa cells after being transfected with a negative control siRNA or two TSC22D1-specific siRNAs.





**Fig. 18** GAPDH protein expression levels in SiHa cells after being transfected with a negative control siRNA or two TSC22D1-specific siRNAs.





**Fig. 19** TSC22D1 protein expression levels in CaSki cells after being transfected with a negative control siRNA or two TSC22D1-specific siRNAs.





**Fig. 20** GAPDH protein expression levels in SiHa cells after being transfected with a negative control siRNA or two TSC22D1-specific siRNAs.





**Fig. 21** E7 protein leves were analyzed by Western blot in SiHa cells underwent transfection with si-E7 and si-NC.





**Fig. 22** PRb protein leves were analyzed by Western blot in SiHa cells underwent transfection with si-E7 and si-NC.





**Fig. 23** MEX3D protein leves were analyzed by Western blot in SiHa cells underwent transfection with si-E7 and si-NC.





**Fig. 24** GAPDH protein leves in SiHa cells underwent transfection with si-E7 and si-NC.





**Fig. 25** E7 protein leves in CaSki cells underwent transfection with si-E7 and si-NC.





**Fig. 26** PRb protein leves in CaSki cells underwent transfection with si-E7 and si-NC.





**Fig. 27** MEX3D protein leves in CaSki cells underwent transfection with si-E7 and si-NC.





**Fig. 28** GAPDH protein leves in CaSki cells underwent transfection with si-E7 and si-NC.
